# Predictive value of clinical indicators in children with community-acquired pneumonia complicated with Kawasaki disease

**DOI:** 10.1016/j.jped.2025.101424

**Published:** 2025-08-12

**Authors:** Yuanhui Duan, Yuexu Ou, Xiaoming Gan, Jieling Li, Jie Cao

**Affiliations:** Department of Medical General Ward Children’s Hospital of Chongqing Medical University, National Clinical Research Center for Child Health and Disorders, Ministry of Education Key Laboratory of Child Development and Disorders, Chongqing Key Laboratory of Child Rare Diseases in Infection and Immunity, Chongqing, China

**Keywords:** Community-acquired pneumonia, Kawasaki disease, Cytokines

## Abstract

**Objective:**

This study aimed to investigate the predictive value of clinical indicators in community-acquired pneumonia (CAP) complicated with Kawasaki disease (KD).

**Methods:**

A retrospective analysis was conducted on the clinical data of inpatients with KD (39 cases), CAP (40 cases), and CAP complicated with KD (CAPKD, 32 cases) at the Children's Hospital of Chongqing Medical University from February 2021 to October 2022. The clinical indicators examined included serum cytokines (IL-2, IL-4, IL-6, IL-10, IL-17A, TNF-α, IFN-γ), White Blood Cell (WBC), Neutrophilic granulocyte percentage(NEU%), blood platelet(PLT), Red Blood Cell (RBC), Hemoglobin(Hb), erythrocyte sedimentation rate(ESR), C-reactive protein(CRP), procalcitonin(PCT), alanine aminotransferase(ALT), alkaline phosphatase(ALP), Gamma-glutamyl transferase (γ-GT), Aspartate aminotransferase (AST), albumin(Alb), Lactate dehydrogenase (LDH), globulin(Glb), and Total Protein(TP) in patients with KD, CAP, and CAPKD were compared.

**Results:**

The present findings showed that IL-6 > 55.4pg/mL, IL-10 > 9.15pg/mL, PCT > 0.19ng/mL, and ALT > 22.5 U/L were important predictors of CAPKD. Additionally, Hb > 103.5 g /L, and TP > 63.85 g/L have predictive values for CAP without KD. The authors also observed a positive correlation between PCT and IL-6, IL-10. However, Hb and TP were negatively correlated with IL-6 and IL-10.

**Conclusion:**

From the perspective of cytokine levels, IL-6 > 55.4 pg/mL and IL-10 > 9.15 pg/mL have important predictive values for CAPKD.

## Introduction

Kawasaki disease (KD) is an acute self-limiting vasculitic disease that can lead to coronary artery lesions (CALs), including coronary artery dilatation (CAD) and coronary artery aneurysm (CAA). It is the most common cause of acquired heart disease in developed countries [1,2]. KD was first described by Dr. Tomisaku Kawasaki in 1967, and mostly affects children under the age of 5 years [3]. While the pathogenesis of KD is still not fully understood, previous studies have found that approximately 35 % of patients may be associated with seasonal infections [4]. It is believed to be an interplay of genetic susceptibility and infectious triggers [5]. KD is primarily diagnosed based on a combination of clinical signs and symptoms [6]. However, Partial KD can present atypically, with incomplete KD accounting for 16.1 % of all KD cases [7]. Therefore, it is crucial to identify laboratory indicators that can predict KD. In past research, KD was found in a small number of children with community-acquired pneumonia (CAP) [8]. CAP is also a prevalent infectious disease and a leading cause of death among children under the age of 5. In practice, the diagnosis of KD, especially incomplete KD, is often missed in patients with CAP. To prevent misdiagnosis and delayed treatment, the present study aims to analyze the differences in clinical characteristics and laboratory data among KD, CAP, and CAPKD patients, and further identify potential predictive indicators for CAPKD.

## Materials and methods

Patients with KD (including complete KD and incomplete KD), CAP, and CAPKD were retrospectively recruited at the Children's Hospital of Chongqing Medical University from February 2021 to October 2022. This study was approved by the Ethics Committee of Children's Hospital of Chongqing Medical University, with the approval number (291) in 2024. All data were collected from medical records, which included demographic information, cardiac ultrasound, chest imaging, and laboratory data, including IL-2, IL-4, IL-6, IL-10, IL-17A, TNF-α, IFN-γ, White Blood Cell (WBC), Neutrophilic granulocyte percentage (NEU %), blood platelet (PLT), Red Blood Cell (RBC), hemoglobin (Hb), erythrocyte sedimentation rate (ESR), C-reactive protein (CRP), procalcitonin (PCT), alanine aminotransferase (ALT), alkaline phosphatase (ALP), Gamma-glutamyl transferase (γ-GT), Aspartate aminotransferase (AST), albumin (Alb), Lactate dehydrogenase (LDH), globulin (Glb), and Total Protein (TP).

### Definition of serum cytokine levels

Venous blood samples were collected to detect Th1/Th2 cytokine levels, including IL-2, IL-4, IL-6, IL-10, IL-17A, TNF-α, and IFN-γ, using cytometric bead assay (CBA). The normal range of these cytokines were (0∼9.80), (0∼3.00), (0∼16.60), (0∼4.90), (0∼14.80), (0∼5.20), and (0∼17.30) pg/mL, respectively.

### Inclusion criteria

1) The diagnosis of KD was made according to guidelines for the diagnosis and treatment of KD in children [6]. Complete KD is diagnosed in the presence of fever for at least 5 days, along with at least 4 of the following 5 main features: a. Erythema and cracking of lips, strawberry-tongue. b. Bilateral bulbar conjunctival Bloodshot without exudate. c. Rash. d. Redness and swelling of the hands and feet in the acute phase and/or periungual desquamation in the subacute phase. e. Cervical lymphadenopathy, usually unilateral. Incomplete KD is diagnosed when an infant presents with prolonged unexplained fever, fewer than four of the above main symptoms, and relevant laboratory or echocardiographic findings.
2) The diagnosis of CAP was made according to diagnostic criteria for community-acquired pneumonia in children [9]. CAP is defined as a clinical diagnosis of pneumonia caused by a community-acquired infection. All patients with CAP had evidence of pneumonia on chest imaging and were tested positively for a respiratory etiology.

### Exclusion criteria

1) patients with immune deficiency, 2) Patients with hematologic tumors, and 3) patients with infection of other systems and organs.

### Detection of respiratory pathogenic microorganisms for CAP

Respiratory pathogenic microorganisms were detected using nasopharyngeal swabs or sputum secretions as specimens. Detection of Respiratory Bacteria: Bacterial cultures and real-time PCR were used to detect the nucleic acid of respiratory pathogens. Detection of Respiratory virus: PCR capillary electrophoresis and immunofluorescence assays were conducted to detect the respiratory virus.

#### Definition of positive respiratory pathogen

The positive results of the specimen culture or bacterial real-time PCR indicated the corresponding bacterial infection and the positive results of the specimens' viral antigen test or DNA PCR indicated the corresponding viral infection.

### Group definition

1) KD group: All children in this group met the diagnostic criteria for KD, and there was no clinical evidence of pneumonia.
2) CAP group: All children in this group met the diagnostic criteria for CAP in children. Chest X-ray or chest CT consistent with the presentation of pneumonia, and positive for respiratory pathogens. All children in this group were examined with cardiac ultrasound, and all children didn’t meet the diagnostic criteria for KD.
3) CAPKD group: All children in this group met the diagnostic criteria for either complete or incomplete Kawasaki disease, as well as for community-acquired pneumonia. They showed evidence of pneumonia on chest X-rays or chest CT scans, along with the presence of positive respiratory pathogens.

### Statistical analysis

SPSS 26 software and GraphPad 9.5 software were employed for statistical analyses. Measurement data were expressed as the median (P25, P75), whereas count data were expressed as the number of cases (percentage). Group comparisons of measurement data following a normal distribution were performed using the independent sample *t*-test, while measurement data with a non-normal distribution were compared using the Wilcoxon rank-sum test. The Kruskal-Wallis test was used for comparison between multiple groups. The Bonferroni correction was applied for multiple comparisons. The receiver operating characteristic (ROC) curve was used to evaluate the predictive value of clinical indicators for CAP complicated with KD. The Spearman correlation was used to evaluate the correlation analysis. Two-sided p values of < 0.05 were considered to indicate statistical significance.

## Results

**Figure 1:**
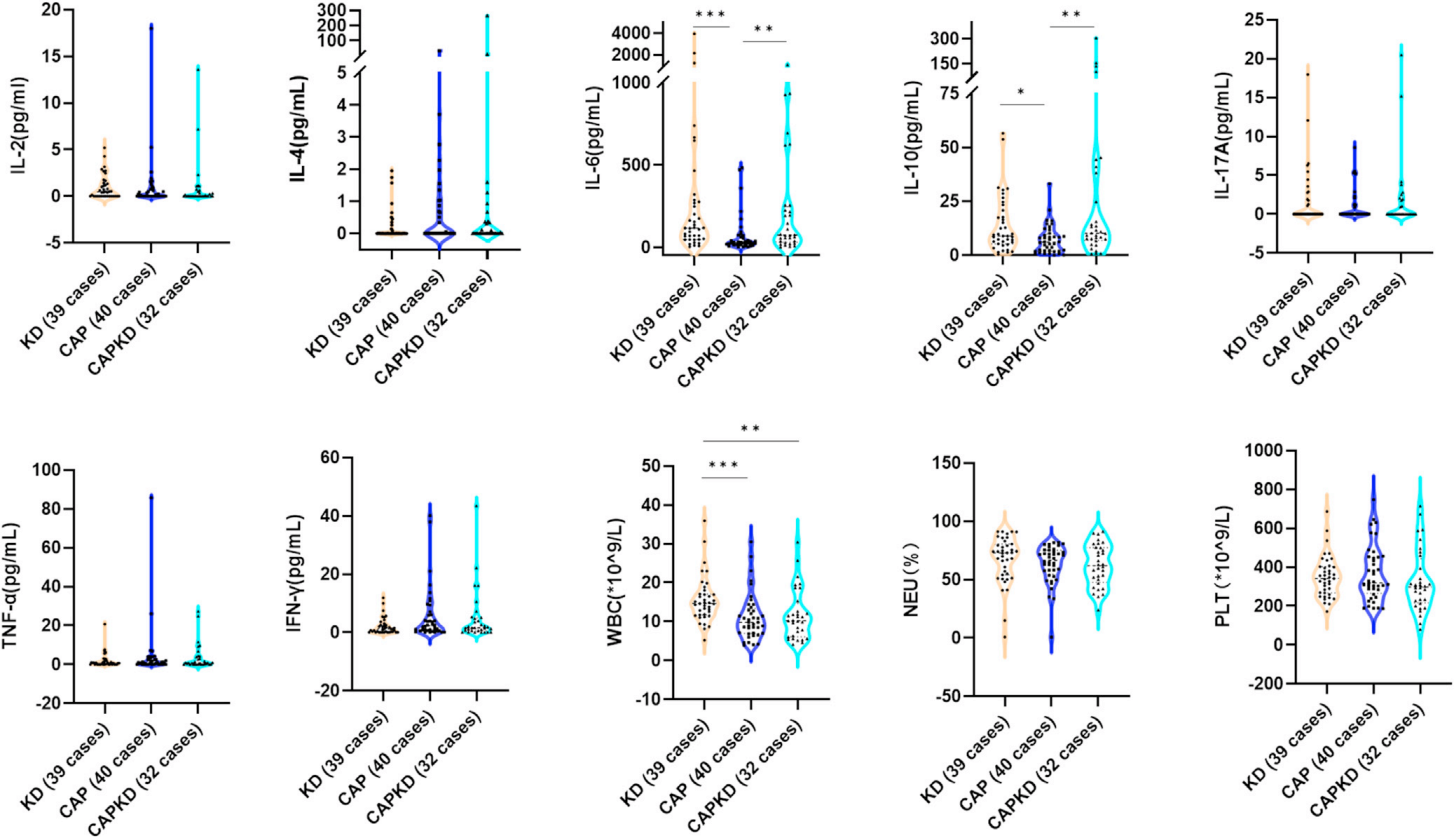
The levels of serum IL-2, IL-4, IL-6, IL-10, IL-17A, TNF-α, IFN-γ and WBC, NEU( %), PLT were compared among the KD group, CAP group, and CAPKD group. IL-2, Interleukin-2; IL-4, Interleukin-4; IL-6, Interleukin-6; IL-10, Interleukin-10; IL-17A, Interleukin-17A; TNF-α, Tumour Necrosis Factor-alpha; IFN-γ, Interferon-gamma; WBC, White Blood Cell; NEU( %), Neutrophilic granulocyte percentage; PLT, platelet; KD, Kawasaki disease; CAP, community-acquired pneumonia; CAPKD, community-acquired pneumonia complicated with Kawasaki disease. * indicate p < 0.05; ** indicate p < 0.01; *** indicate p <0.001.

**Figure 2:**
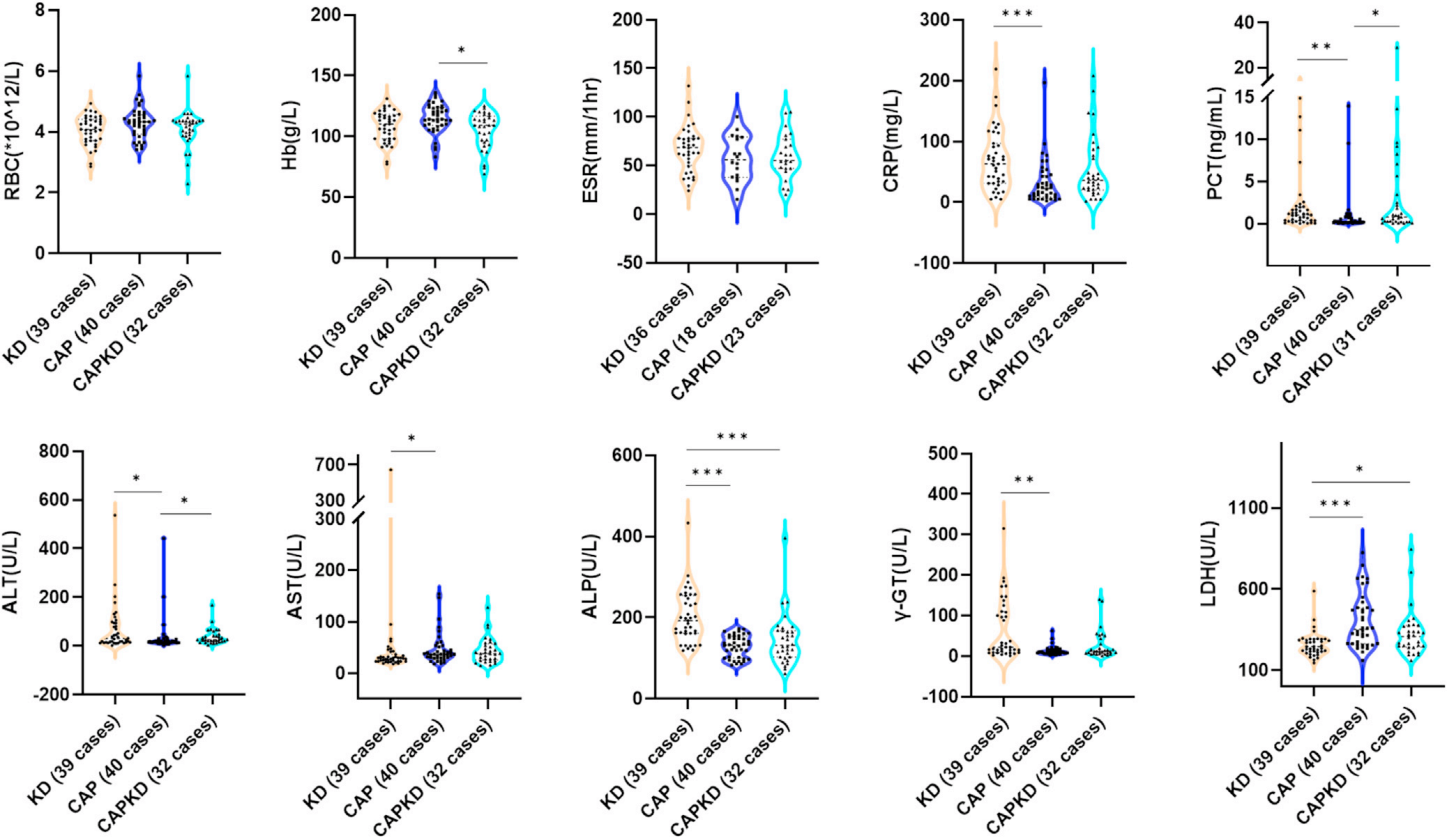
The levels of serum Levels of RBC, Hb, ESR, CRP, PCT, ALT, AST, ALP, γ-GT, and LDH were compared among the KD group, CAP group and CAPKD group. RBC, Red Blood Cell; Hb, hemoglobin; ESR, erythrocyte sedimentation rate; CRP, C-reactive protein; PCT, procalcitonin; ALT, alanine aminotransferase; ALP, alkaline phosphatase; γ-GT, Gamma-glutamyltransferase; AST, Aspartate aminotransferase; Alb, albumin; LDH, Lactate dehydrogenase; Glb, globulin; TP, Total Protein; KD, Kawasaki disease; CAP, community-acquired pneumonia; CAPKD, community-acquired pneumonia complicated with Kawasaki disease. * indicate p < 0.05; ** indicate p < 0.01; *** indicate p < 0.001.

**Figure 3:**
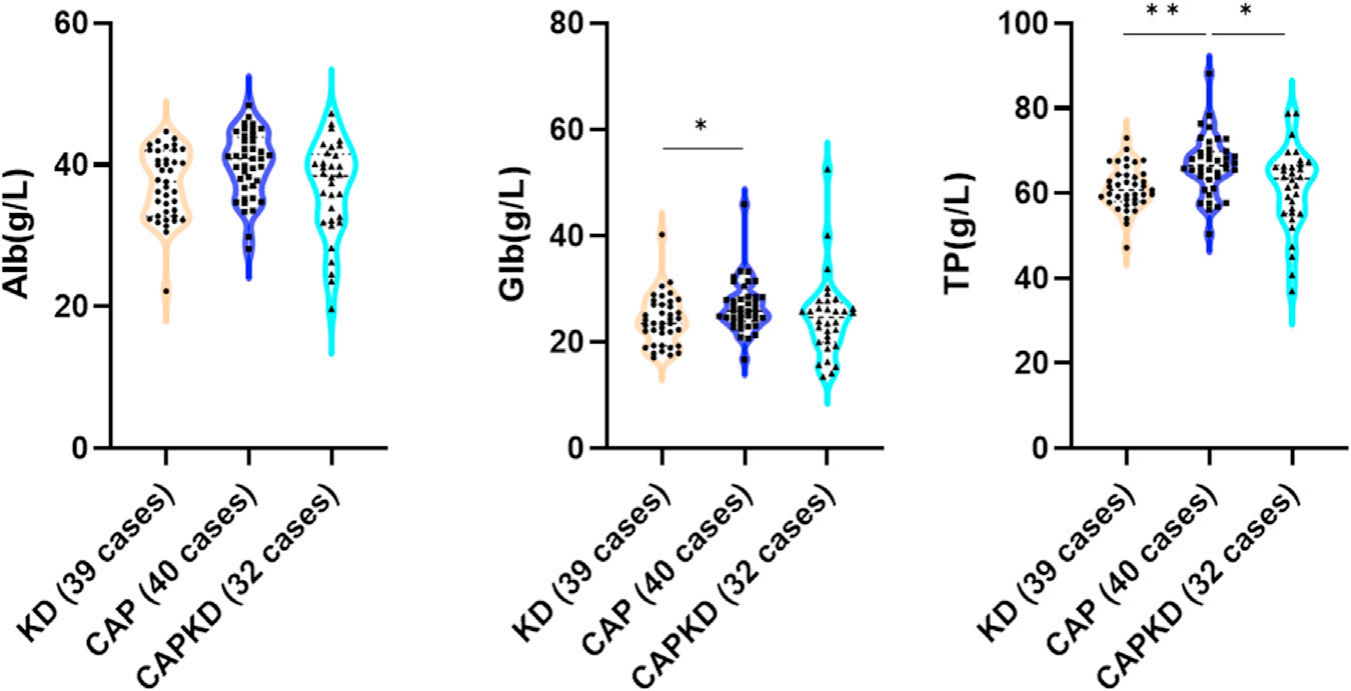
The levels of serum Levels of Alb, Glb, and TP were compared among the KD group, CAP group, and CAPKD group. Alb, albumin; Glb, globulin; TP, Total Protein; KD, Kawasaki disease; CAP, community-acquired pneumonia; CAPKD; community-acquired pneumonia complicated with Kawasaki disease. * indicate p <0.05; ** indicate p <0.01; *** indicate p < 0.001.

A total of 111 patients were included in this study, with 39 cases in the KD group, 40 cases in the CAP group, and 32 cases in the CAPKD group (the screening process can be found in supplementary material, Flow chart 1). The male-to-female ratio in the KD group was 1.44:1, 1:1.22 in the CAP group, and 2.56:1 in the CAPKD group, listed in Supplementary Material Table 1. This suggests that the KD and CAPKD groups had a higher proportion of males than the CAP group. In terms of age, the median ages (P25, P75) were 2.58 years (1.67, 4.79) in the KD group and 2.17 years (1.5, 3.17) in the CAPKD group, which were both lower than the median age of 3.5 years (2.11, 6.92) in the CAP group (*p* = 0.016). The fever time in admission was significantly longer in the CAP group [8 (6.75,10.25) days] than in the KD group [5 (4,6) days, p <0.001] and in the CAPKD group [6 (4,7) days, *p* < 0.01]. However, the present study further found that the fever time before the ultrasonic cardiogram was significantly longer in the CAPKD group [7 (5,8) days] than in the KD group [5 (4,6) days], which implies that the delayed detection of coronary artery lesions in the CAPKD group. There were significant differences in clinical indicators between the KD, CAP, and CAPKD groups. Further details can be found in Figure 1, Figure 2, Figure 3 (and Supplementary Material Table 1). The present study showed that various respiratory pathogens can cause CAPKD, mainly including 10 cases of *Haemophilus influenzae*, 9 cases of *Respiratory syncytial virus*, and 8 cases of *Streptococcus pneumoniae* (detailed in Supplementary Material Table 2).

Compared with the CAPKD group, the levels of WBC and ALP were significantly higher in the KD group (p<0.05). While the level of LDH was lower in the KD group than in the CAPKD group (p<0.05). No statistical difference was observed in the levels of IL‐2, IL‐4, IL‐6, IL‐10, IL‐17A, TNF‐α, IFN‐γ, NEU %, PLT, RBC, Hb, ESR, CRP, PCT, ALT, AST, γ-GT, Alb, Glb, and TP between the KD group and the CAPKD group. This result indicates that the patients of CAPKD group in the present study are good representatives of KD.

Compared with the CAP group, the levels of IL-6, IL-10, WBC, CRP, PCT, ALT, ALP, and γ-GT were significantly higher in the KD group (p<0.05). While the levels of AST, LDH, Glb, and TP were lower in the KD group than in the CAP group (p<0.05). No statistical difference was observed in the levels of IL‐2, IL‐4, IL‐17A, TNF‐α, IFN‐γ, NEU %, PLT, RBC, Hb, ESR, γ-GT, and Alb between the KD group and the CAP group.

Compared with the CAP group, the levels of IL-6, IL-10, PCT, and ALT were significantly higher in the CAPKD group (p<0.05). While the levels of Hb and TP were lower in the CAPKD group than in the CAP group (p<0.05). No statistical difference was observed in the levels of IL‐2, IL‐4, IL‐17A, TNF‐α, IFN‐γ, WBC, NEU %, PLT, RBC, ESR, CRP, ALP, AST, γ-GT, LDH, Alb, and Glb between the CAPKD group and the CAP group.

**Figure 4:**
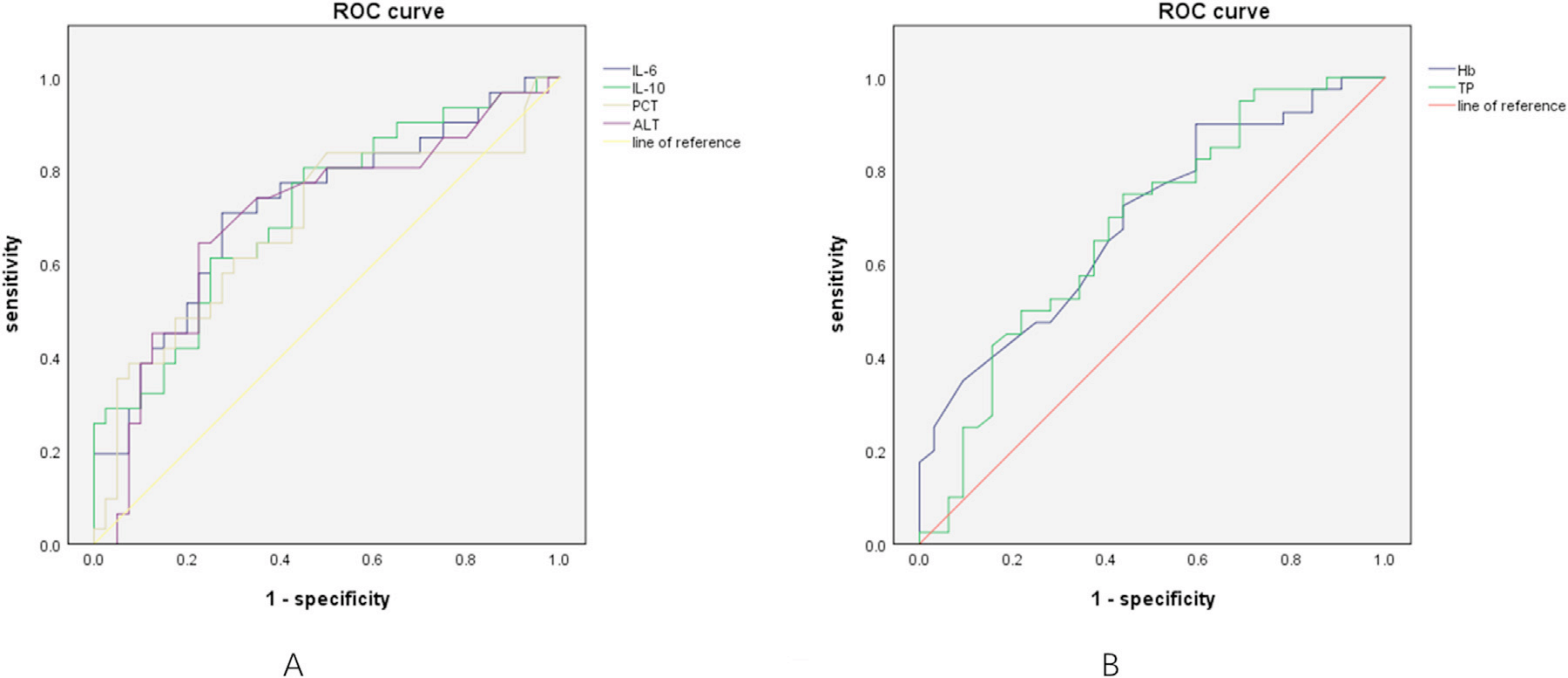
(A) The predictive value of IL-6,IL-10,PCT,ALT in CAP complicated with KD. (B) The predictive value of Hb,TP in CAP not complicated with KD. ROC, receiver operating characteristic; IL-6, Interleukin-6; IL-10, Interleukin-10; PCT, procalcitonin; ALT, alanine aminotransferase; Hb, hemoglobin; TP, Total Protein; KD, Kawasaki disease; CAP, community-acquired pneumonia.

Additionally, according to the ROC curve analysis, the authors further investigated the predictive value of these indicators in CAPKD or CAP without KD (Figure 4A and 4B). The elevated levels of IL-6, IL-10, PCT, and ALT can be used as predictors of CAPKD, with an area under the ROC curve of 0.722 (95 % CI: 0.600, 0.843, *p* < 0.01; cut-off value 55.4), 0.714 (95 % CI: 0.593, 0.835, *p* < 0.01, cut-off value 9.15), 0.682 (95 % CI: 0.551, 0.813, *p* < 0.01, cut-off value 0.19), and 0.7 (95 % CI: 0.573, 0.828, *p* < 0.01, cut-off value 22.5), respectively (Supplementary Material Table 3). Conversely, the elevated levels of Hb and TP can be used as predictors of CAP without KD, with an area under the ROC curve of 0.696 (95 % CI: 0.574, 0.818, *p* < 0.01; cut-off value 103.5), and 0.699 (95 % CI: 0.576, 0.823, *p* < 0.01, cut-off value 63.85), respectively (Supplementary Material Table 4).

The present study showed that the level of serum IL-6 was positively correlated with CRP, PCT, and γ-GT (*r* = 0.506, 0.416, and 0.204, respectively, with p<0.05). Inversely, the level of serum IL-6 was negatively correlated with Hb, Alb, Glb, and TP (*r* = −0.281, −0.308, −0.238, and −0.390, respectively, with p<0.05). Additionally, the authors also found that the level of IL-10 was positively correlated with PCT (*r* = 0.377). Similarly, the level of serum IL-10 was negatively correlated with Hb, Glb, and TP (*r* = −0.213, −0.340, and −0.389, respectively, with p<0.05). The correlation analysis between IL-6, IL-10, and other clinical indicators (Detailed in Supplementary Material Table 5).

## Discussion

The incidence of Kawasaki disease (KD) varies across different regions, with a higher prevalence among Asian children and a male-to-female ratio of approximately 1.5:1 [6]. The present findings also showed a high proportion of males in both the KD and CAPKD groups. The exact cause of KD is still unknown, but it is currently believed to be an acute systemic immune vasculitis triggered by infection in genetically predisposed individuals [10]. CAP is a common infectious disease in children. Due to the global prevalence of SARS-CoV-2, previous studies have found that Kawasaki disease or Kawasaki-like multisystem inflammatory syndrome was related to infection with SARS-CoV-2. These patients often experience persistent fever, elevated inflammation, and develop coronary artery lesions [11, 12, 13]. Fernanda Falcini reported a case of a 30-month-old child with refractory pneumonia who was complicated with atypical Kawasaki disease with a coronary aneurysm [14]. Yoshiki Kawamura also reported a case of neonatal pneumonia complicated with atypical Kawasaki disease and coronary aneurysm [15]. In clinical practice, CAPKD, especially with atypical CAPKD, is often overlooked and can result in delayed diagnosis and treatment, potentially leading to the development of CAL. Specifically, 9 cases (28.12 %) of children with atypical KD and 15 cases (46.87 %) of children with coronary aneurysm were identified in the CAPKD group. Additionally, the main respiratory pathogens in the CAPKD group were *Haemophilus influenzae, respiratory syncytial virus, Streptococcus pneumoniae, adenovirus, and Mycoplasma pneumoniae*. However, there is a lack of studies that have identified clinical indicators with predictive value for CAPKD.

Cytokines play a crucial role in regulating immune inflammatory responses and in the occurrence and development of diseases [16,17]. Intravenous immunoglobulin (IVIG) is one of the primary therapies used in the acute phase of KD, and it has been shown to modulate the synthesis and release of proinflammatory cytokines [18]. This suggests that cytokines have a significant impact on the development of KD disease. IL-6, as the main pro-inflammatory cytokine, can be produced immediately and contributes to host defense against infection by stimulating specific cellular and humoral immune responses [19]. IL-10, as an anti-inflammatory cytokine, can suppress severe inflammatory responses by inhibiting macrophages and neutrophils, and inhibiting T-cell responses [20]. The present study found that the levels of IL-6 and IL-10 in the KD group and CAPKD group were significantly higher than in the CAP group. This suggests that the inflammatory response in KD and CAPKD is stronger compared to CAP, and the anti-inflammatory response also increased due to the host's inflammatory response feedback. Through ROC curve analysis, the present study first found that IL-6 > 55.4 pg/mL and IL-10 > 9.15 pg/mL have predictive value in CAPKD.

The level of Hb in the CAPKD group was lower than in the CAP group. Hemoglobin is a protein in red blood cells that carries oxygen to the tissues of the human body. The production of hemoglobin is affected by various factors. In cases where the body is experiencing an inflammatory response due to disease, the release of pro-inflammatory factors such as IL-6 can decrease serum iron levels and inhibit the production of red blood cells, resulting in inflammatory anemia [21]. The present study also found a negative correlation between serum IL-6 and IL-10 levels and Hb. Additionally, this study showed that Hb > 103.5 g /L has a significant predictive value for CAP without KD.

Both CRP and PCT are commonly used as indicators of inflammation in clinical practice, as they have predictive value in the development of sepsis. CRP is an acute responsive phase protein produced by the liver, while PCT is typically used to identify sepsis and non-sepsis [22]. In the present study, there was no significant difference in the level of CRP between the CAP group and the CAPKD group, indicating that CRP may not have a clear predictive value for CAPKD. Further studies are needed to confirm this. However, the level of PCT in the CAPKD group was higher than in the CAP group, and there was a positive correlation between IL-6 levels and the elevation of CRP and PCT. This is consistent with previous research showing that IL-6 stimulates the host to produce CRP and PCT [23]. Furthermore, the authors found that PCT > 0.19ng/mL has an important predictive value for CAPKD.

Hepatic function damage is a common complication during the acute phase of KD. This damage is characterized by elevated levels of serum liver enzymes, hypoalbuminemia, and hyperbilirubinemia [24]. The level of Serum ALT in the KD group and CAPKD group was higher than in the CAP group, indicating this finding is consistent with previous research that KD was more likely to cause liver function damage. Additionally, the present study also found that ALT > 22.5 U/L can be a significant predictor of CAPKD. The level of serum TP in the CAP group was higher than in the KD group and the CAPKD group. This could be attributed to the higher inflammatory response in the KD and CAPKD groups, leading to liver function damage and decreased albumin levels. In addition, the inflammatory response also consumes a significant amount of immunoglobulin, resulting in a decrease in globulin levels. According to the ROC curve analysis, TP > 63.85 g/L has a predictive value for CAP without KD. Additionally, the authors found that the level of LDH in the KD group was lower than in the CAP group and the CAPKD group. This suggests that patients with pulmonary infection have higher LDH levels compared to those without pulmonary infection, which is consistent with previous studies [25]. It indicated that LDH level can be used as a predictor of the KD patients complicated with pneumonia, but it needs further investigations.

However, the present study still has some limitations that should be noted. Firstly, it is a retrospective study, which may have resulted in incomplete information or selection and information bias. Additionally, the sample size for each group of patients was small due to the strict screening criteria. Furthermore, this study was conducted at a single center, and more multi-center studies are needed to confirm the present findings.

## Conclusion

The present study first analyzed the clinical predictive indicators of CAPKD. The authors found that IL-6 > 55.4pg/mL, IL-10 > 9.15pg/mL, PCT > 0.19ng/mL, and ALT > 22.5 U/L have significant predictive values for CAPKD. Hb > 103.5 g /L and TP > 63.85 g/L have important predictive values for CAP without KD. In addition, the correlation analysis showed that PCT was positively correlated with IL-6 and IL-10, while Hb and TP were negatively correlated with IL-6 and IL-10. Therefore, from the perspective of cytokines analysis, IL-6 > 55.4 pg/mL and IL-10 > 9.15 pg/mL can be used as important predictors of CAPKD.

## Consent statement

This study was performed in line with the principles of the Declaration of Helsinki and was approved by the Ethics Committee of Children's Hospital of Chongqing Medical University, with approval number (291) in 2024. Informed consent was obtained from the parents or legal guardians in written form when children were admitted to the hospital.

## Authors’ contributions

Y.D. collected the data and drafted the article. Y.O., J.L., and X.G. conducted data curation and investigation. J.C. Designed and reviewed study. All authors have approved the final manuscript as submitted and agree to be responsible for all aspects of the work.

## Conflicts of interest

The authors declare no conflicts of interest with respect to the research, authorship, and/or publication of this article.

## Appendix Supplementary materials

Image, application 1

## Footnotes

Supplementary material associated with this article can be found in the online version at doi:10.1016/j.jped.2025.101424.
