# Supplementary material for: Predictive value of clinical indicators in children with community-acquired pneumonia complicated with Kawasaki disease
Source: J Pediatr (Rio J). 2025 Aug 12;101(5):101424. doi: 10.1016/j.jped.2025.101424 (PMC12361764; doi:10.1016/j.jped.2025.101424)
Supplement: Supplementary file 1 [file mmc1.docx]

**JPED-D-25-00082 SupplementaryMaterials**


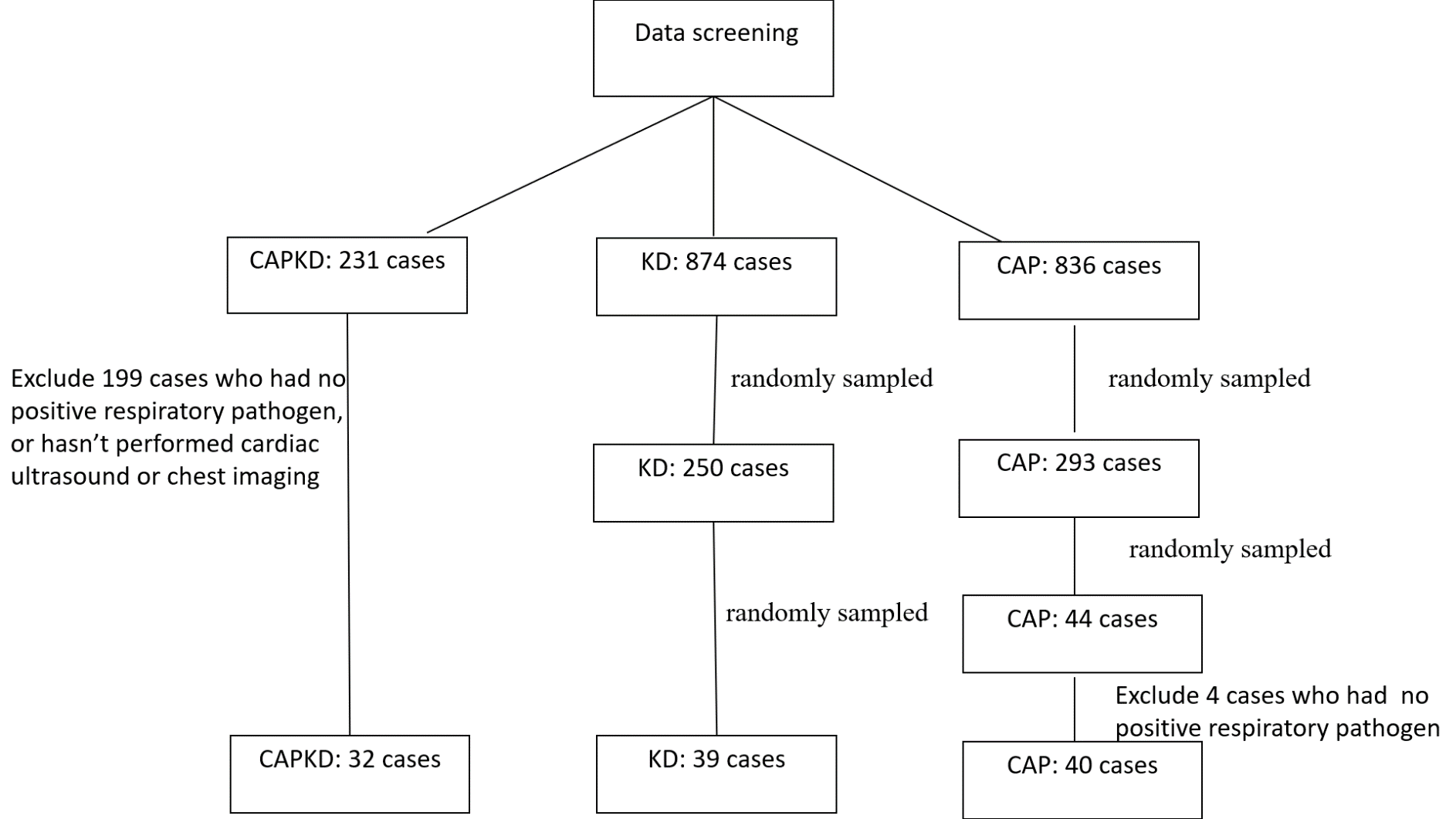


**Flow chart 1** A flow chart presents the screening process of CAPKD, KD, and CAP patients.

**Supplementary Table 1** Summary of basic characteristics and clinical indicators among different groups.

|  | KD group（39 cases） | CAP group（40 cases） | CAPKD group（32 cases） | P |
| --- | --- | --- | --- | --- |
| Gender |  |  |  |  |
| Male | 23 | 18 | 23 | 0.071 |
| Female | 16 | 22 | 9 |  |
| Age | 2.58(1.67,4.79) | 3.5(2.11,6.92) | 2.17(1.5,3.17) | 0.016* |
| Fever time in admission (days) | 5(4,6) | 8(6.75,10.25) | 6(4,7) | 0*** |
| Fever time before ultrasonic cardiogram (days) | 5(4,6) |  | 7(5,8) | 0.021* |
| Complete KD | 35 |  | 23 |  |
| incomplete KD | 4 |  | 9 |  |
| coronary artery aneurysm | 14 |  | 15 |  |
| No coronary artery aneurysm | 25 |  | 17 |  |
| IL-2 | 0.42(0.005,1.55) | 0.11(0,0.64) | 0.09(0,0.85) | 0.104 |
| IL-4 | 0(0,0.11) | 0(0,0.59) | 0(0,0.51) | 0.47 |
| IL-6 | 117.65(47.53,258.64) | 23.35(14.3,43.75) | 72.05(26.76,220) | 0*** |
| IL-10 | 8.49(4.72,16.37) | 5.73(1.74,9.46) | 10.2(7.04,43.7) | 0.005** |
| IL-17A | 0.01(0,1.84) | 0(0,1.18) | 0.01(0,2.64) | 0.36 |
| TNF-α | 0.65(0.02,1.64) | 0.47(0,2.97) | 0.8(0.21,5.58) | 0.648 |
| IFN-γ | 0.69(0.01,2.34) | 2.68(0.56,5.86) | 1.95(0.66,6.27) | 0.093 |
| WBC count(*10^9/L) | 14.59(11.79,16.71) | 11.14(8.16,14.67) | 10.19(6.16,11.84) | 0*** |
| neutrophil percentage(%) | 68(51.1,79.2) | 66.6(49.5,80.05) | 61(49.35,75.4) | 0.272 |
| PLT(*10^9/L,) | 347(276,404) | 311(297,461) | 308(249,468) | 0.305 |
| RBC(*10^12/L) | 4.14(3.74,4.47) | 4.32(3.61,4.58) | 4.29(3.86,4.42) | 0.168 |
| Hb(g/L) | 110(98,117.5) | 118(102.25,125.25) | 109(96,116) | 0.01* |
| ESR | 64(46,78) | 56(37.75,79.25) | 55(47,79) | 0.313 |
| CRP | 61(32.5,91.12) | 22.77(5,48.67) | 33(18.59,60.3) | 0*** |
| PCT | 1.02(0.34,1.82) | 0.29(0.1,0.89) | 0.62(0.24,2) | 0.002** |
| ALT | 36(16,94) | 18(13.75,28.5) | 33.4(18,54.5) | 0.006** |
| AST | 30(23,39.5) | 39.5(34.5,58.25) | 38.3(23.5,60) | 0.019* |
| ALP | 185(161,241) | 127.5(101,152) | 150(101.5,175.5) | 0*** |
| γ-GT | 22(13.5,109) | 11(6,16.5) | 15.3(7.5,52) | 0.002** |
| LDH | 250(216.45,292.5) | 343.5(256.5,642.25) | 275(240.5,374) | 0*** |
| Alb | 39.2(34.05,42.05) | 41.25(36.4,42.75) | 36(31.8,42.05) | 0.026* |
| Glb | 23.5(19.3,27) | 24.75(23.23,28.1) | 23.4(20,27.85) | 0.024* |
| TP | 60.8(58.2,64.45) | 65.25(60.48,68.93) | 61.8(55.15,67) | 0.001** |

Note: * indicate p ＜0.05; ** indicate p＜0.01;*** indicate p＜0.001

**Supplementary Table 2** Positive cases of respiratory pathogens in the CAP group and the CAPKD group.

|  | CAP group (cases) | CAPKD group (cases) |
| --- | --- | --- |
| Respiratory pathogens |  |  |
| Streptococcus pneumoniae | 7 | 8 |
| Haemophilus influenzae | 11 | 10 |
| Moraxella catarrata | 1 | 1 |
| Mycoplasma pneumoniae | 21 | 3 |
| Adenovirus | 6 | 4 |
| Respiratory syncytial virus | 5 | 9 |
| Influenza B virus | 0 | 1 |
| Parainfluenza virus | 0 | 2 |

**Supplementary Table 3** Clinical indicators for predicting CAP complicated with KD.

|  | AUC | P | AUC 95%CI |  | Youden index maximum | cutoff value | sensitivity | Specificity |
| --- | --- | --- | --- | --- | --- | --- | --- | --- |
| IL-6 | 0.722 | 0.001 | 0.600，0.843 | | 0.435 | 55.4 | 0.71 | 0.725 |
| IL-10 | 0.714 | 0.002 | 0.593，0.835 | | 0.363 | 9.15 | 0.613 | 0.75 |
| PCT | 0.682 | 0.009 | 0.551，0.813 | | 0.339 | 0.19 | 0.839 | 0.5 |
| ALT | 0.700 | 0.004 | 0.573，0.828 | | 0.42 | 22.5 | 0.645 | 0.775 |

**Supplementary Table 4** Clinical indicators for predicting CAP not complicated with KD.

|  | AUC | P | AUC 95%CI | Youden index maximum | cutoff value | Sensitivity | Specificity |
| --- | --- | --- | --- | --- | --- | --- | --- |
| Hb | 0.696 | 0.005 | 0.574，0.818 | 0.319 | 103.5 | 0.9 | 0.419 |
| TP | 0.699 | 0.004 | 0.576，0.823 | 0.331 | 63.85 | 0.75 | 0.58 |

**Supplementary Table 5** The correlation analysis between IL-6, IL-10 and other clinical indicator.

|  |  | WBC | Hb | CRP | PCT | ALT | AST | ALP | γ-GT | LDH | Alb | Glb | TP |
| --- | --- | --- | --- | --- | --- | --- | --- | --- | --- | --- | --- | --- | --- |
| IL-6 | r | 0.157 | -0.281 | 0.506 | 0.416 | 0.181 | -0.089 | 0.097 | 0.204 | -0.113 | -0.308 | -0.238 | -0.390 |
|  | P | 0.099 | 0.003 | 0.000 | 0.000 | 0.057 | 0.355 | 0.314 | 0.037 | 0.238 | 0.001 | 0.016 | 0.000 |
| IL-10 | r | 0.065 | -0.213 | 0.139 | 0.377 | 0.157 | 0.075 | 0.039 | 0.108 | 0.033 | -0.170 | -0.340 | -0.389 |
|  | P | 0.499 | 0.025 | 0.145 | 0.000 | 0.099 | 0.437 | 0.683 | 0.273 | 0.734 | 0.075 | 0.000 | 0.000 |
